# Identification of Biofilm-Associated Cluster (*bac*) in *Pseudomonas aeruginosa* Involved in Biofilm Formation and Virulence

**DOI:** 10.1371/journal.pone.0003897

**Published:** 2008-12-09

**Authors:** Camille Macé, Damien Seyer, Chanez Chemani, Pascal Cosette, Patrick Di-Martino, Benoit Guery, Alain Filloux, Marc Fontaine, Virginie Molle, Guy-Alain Junter, Thierry Jouenne

**Affiliations:** 1 Université de Rouen, Laboratoire “Polymères, Biopolymères, Surfaces”, CNRS FRE 3101, Plate-forme protéomique de l'IFRMP 23, Mont-Saint-Aignan, France; 2 Laboratoire ERRMECe (EA1391), Université de Cergy-Pontoise, Pontoise, France; 3 Laboratoire 〈〈 Détresses Respiratoires et Circulatoires. Physiopathologie, mécanismes d'adaptation 〉〉 (EA 2689), Université de Lille 2, Lille, France; 4 Centre for Molecular Microbiology and Infection, Faculty of Natural Sciences, Imperial College London, London, United Kingdom; 5 Université de Rouen, EA 4130, Mont-Saint-Aignan, France; 6 CNRS, UMR 5086, Institut de Biologie et Chimie des Protéines, Lyon, France; Texas Tech University Health Sciences Center, United States of America

## Abstract

Biofilms are prevalent in diseases caused by *Pseudomonas aeruginosa*, an opportunistic and nosocomial pathogen. By a proteomic approach, we previously identified a hypothetical protein of *P. aeruginosa* (coded by the gene *pA3731*) that was accumulated by biofilm cells. We report here that a Δ*pA3731* mutant is highly biofilm-defective as compared with the wild-type strain. Using a mouse model of lung infection, we show that the mutation also induces a defect in bacterial growth during the acute phase of infection and an attenuation of the virulence. The *pA3731* gene is found to control positively the ability to swarm and to produce extracellular rhamnolipids, and belongs to a cluster of 4 genes (*pA3729–pA3732*) not previously described in *P. aeruginosa*. Though the protein PA3731 has a predicted secondary structure similar to that of the Phage Shock Protein, some obvious differences are observed compared to already described *psp* systems, e.g., this unknown cluster is monocistronic and no homology is found between the other proteins constituting this locus and *psp* proteins. As *E. coli* PspA, the amount of the protein PA3731 is enlarged by an osmotic shock, however, not affected by a heat shock. We consequently named this locus *bac* for biofilm-associated cluster.

## Introduction

In most environments, bacteria predominantly grow in association with surfaces, leading to the formation of biofilms [1]. Bacterial attachment to surfaces and the subsequent biofilm formation are important steps in the establishment of chronic infections and persistence in host tissues [1]. *Pseudomonas aeruginosa*, an opportunistic Gram-negative pathogen, is able to attach to abiotic and biotic surfaces. *P. aeruginosa* biofilms are important issues in the pathogenesis of the bacterium in ventilator-associated pneumonia, urinary and peritoneal dialysis catheter infections, bacterial keratitis, otitis externa and burn wound infections [1]. Chronic lung infection by *P. aeruginosa* leads to a decline of lung function, respiratory failure, and ultimately, death in cystic fibrosis (CF) patients [2]. The mechanisms involved in bacterial adhesion have been increasingly investigated over the last decade. Flagella and type IV pili [3], Cup fimbria [4] and *pel* genes [5] are the most frequently cited *P. aeruginosa* determinants among those shown to be implicated at various stages of biofilm formation. Using a proteomic approach, we previously identified many proteins up-regulated in sessile *P. aeruginosa* cells [6], among which was the PA3731 protein. To evaluate the role of this protein in the biofilm formation, we compare here the ability of the wild-type strain and Δ*pA3731* mutant to adhere on abiotic and biotic surfaces. Results demonstrate that the mutant is highly biofilm-defective. Experiments performed using a mouse model of lung infection show that the mutant exhibits a defect in bacterial growth during the acute phase of infection and is attenuated for virulence. In addition, the *pA3731* gene is shown to be required for swarming motility and rhamnolipid synthesis. This gene belongs to a cluster of 4 genes (*pA3729*–*pA373*2) of unknown function. The predicted secondary structure of PA3731, largely helical along the entire length of the protein, shares similarities with the Phage shock Protein A (PspA). As for *psp* regulons, we identified a putative RpoN-binding site upstream the *pA3732* gene and as PspA, PA3731 is accumulated by an osmotic shock. However, some obvious differences are observed compared to already described *psp* systems, e.g., this unknown cluster is monocistronic, no homology is found between the other proteins constituting this locus and *psp* proteins and PA3731 is not affected by a temperature shift from 37 to 50°C. These data suggest that this cluster (that we named *bac* for biofilm-associated cluster) even though of unknown function, is a major pathway for biofilm formation and virulence in *P. aeruginosa*.

## Results

### pa3731 is involved in the interaction of P. aeruginosa with abiotic and biotic surfaces

**Figure 1:**
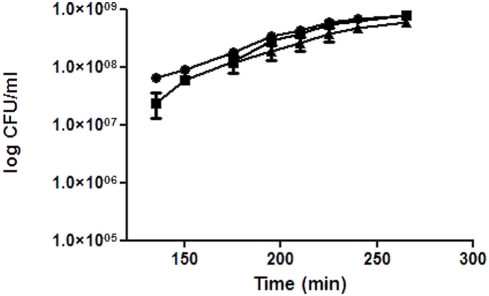
Planktonic growth kinetics of PAO1 (Black circle), *ΔpA3731* (Black square) and *ΔpA3731*_comp_ (Black triangle) strains. Bars: SEM. (n = 3).

**Figure 2:**
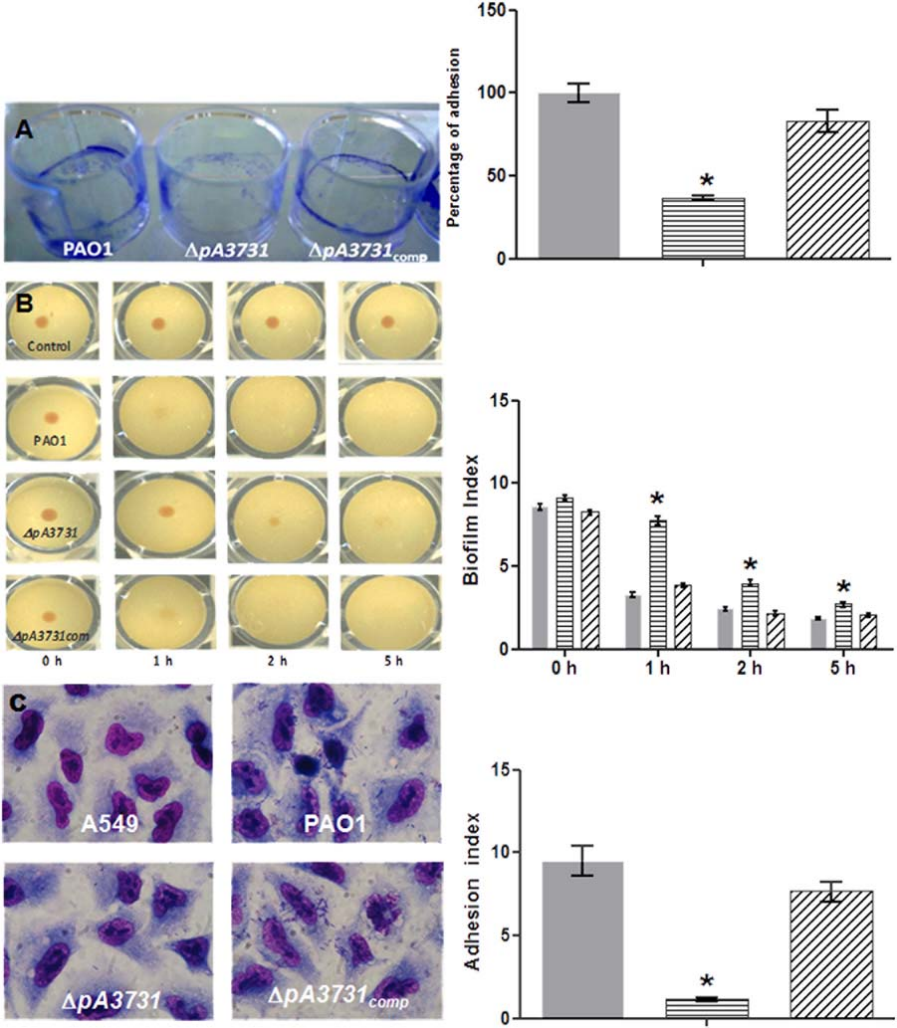
Quantification of biofilm formation by the PAO1, *ΔpA3731* and *ΔpA3731*_comp_ strains using (A) the crystal violet test; (B) the Biofilm Ring Test®, control without bacteria; (C), adherence to human A549 pneumocyte cells (cells and bacteria were stained with 20% Giemsa); A549, control without bacteria; Magnification, ×1000). PAO1 (Grey); *ΔpA3731* (Horizontal line); *ΔpA3731*_comp_ (Oblique line). Bars: SEM (n = 10); *, P≤0.05.

**Figure 3:**
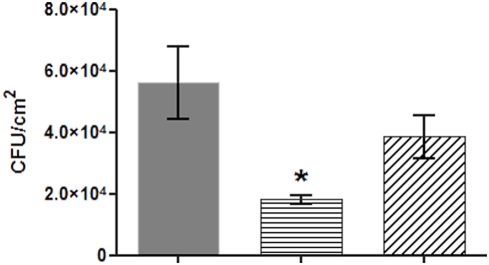
Adhesion assays in PBS. PAO1 (Grey); *ΔpA3731* (Horizontal line); *ΔpA3731*_comp_ (Oblique line). Bars: SEM (n = 10); *, P≤0.05.

Growth kinetics of planktonic cells in LB broth (Figure 1) showed that the *pA3731* mutation did not alter bacterial growth (generation time, 30 min for PAO1 and Δ*pA3731*). The complemented strain (Δ*pA3731_comp_*) grew a little bit more slowly (generation time 35 min). To determine whether the *pA3731*gene is a true biofilm specific factor, we first investigated the influence of the *pA3731* mutation on the ability of *P. aeruginosa* to adhere on polystyrene plastic surfaces. Determination by crystal violet staining (Fig. 2A) revealed a significant (P≤0.05, n = 10) decrease of about 77% of the ring at the air-liquid interface as compared with the wild-type, after incubation for 24 h at 37°C in LB broth, and the mutant was consequently considered as non adherent. This phenotype was then confirmed by using the BioFilm Ring Test ®, i.e., an innovative biofilm assay recently developed [7]. Images obtained with the parent strain and the mutant confirmed the alteration in the biofilm-forming ability of the Δ*pA3731* strain (Fig. 2B). Whereas a biofilm was already formed by the wild-type after 2 h of incubation at 37°C (BioFilm Index (BFI) of 2.4±0.1), a BFI value of 4.0±0.2 was obtained with the mutant. After 5 h of incubation, a significant difference (P≤0.05, n = 10) was still observed between the two strains, BFI values of 1.8±0.1 and 2.7±0.2 being obtained with PAO1 and Δ*pA3731* strains, respectively. The parental strain PAO1 and its Δ*pA3731*mutant were then compared for their ability to adhere on A549 human pneumocyte cells. Microscopic examinations showed that the wild-type strain could adhere diffusely to the cell line, bacteria scattering over the eukaryotic cell surface (Fig. 2C). An adhesion index of 9.5±3.1 was measured. The mutation on the *pA3731*gene made the bacterium unable to adhere on pneumocytes, which is highlighted by the adhesion index of 1.1±0.3 obtained for the mutant. Due to the difficulty to distinguish in the “biofilm formation” what is due to adhesion, growth, production of exopolymères, we evaluated the number of bacteria adhering on polystyrene wells after incubation for 0.5 h in Phosphate Buffered Saline (PBS). Results (Fig. 3) showed a significant (P≤0.05, n = 10) decrease of the number of adherent cells with the mutant as compared with the wild-type. Complementation of the Δ*pA3731* mutant with pMMB67-HE14 expressing *pA3731* restored the wild-type phenotypes

### Bacterial motility

**Figure 4:**
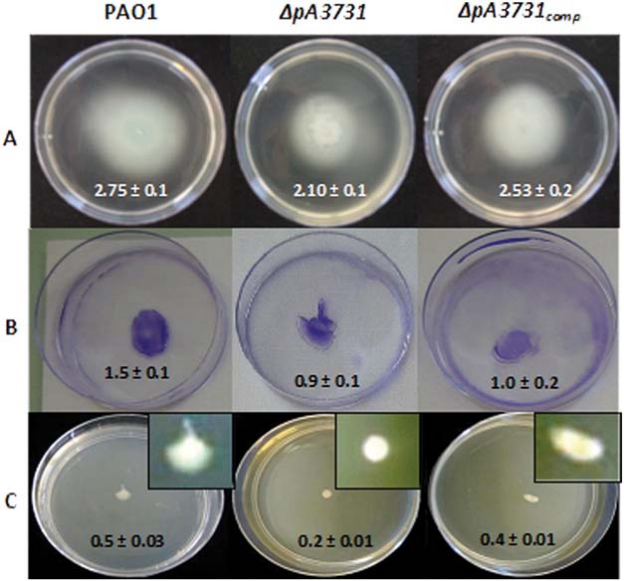
Motility assays of PAO1, *ΔpA3731* and *ΔpA3731*_comp_ strains. Cells were inoculated with a needle into the bottom of LB agar medium. Plates were incubated at 37°C for 24 h. A. swimming; B, twitching; C, swarming (magnification views, ×3).

To evaluate the impact of the mutation on the synthesis or functioning of flagella and type IV pili, we performed swimming, swarming and twitching assays. Macroscopic assays showed that the mutation had no effect on the flagellum-mediated motility (i.e., swimming) (Fig. 4A). The diameters of the migration zones produced by the parental and mutant strains were not significantly different (P>0.05, n = 12). The twitching seemed also not altered (P>0.05, n = 12): the wild-type and mutant strains spread by twitching motility and formed flat colonies (Fig. 4B). In contrast, the mutant failed to spread by swarming motility (Fig. 4C) as the diameter of the mutant migration zone decreased by about 60% as compared with the wild-type (P≤0.05, n = 12).

### Rhamnolipid synthesis

**Figure 5:**
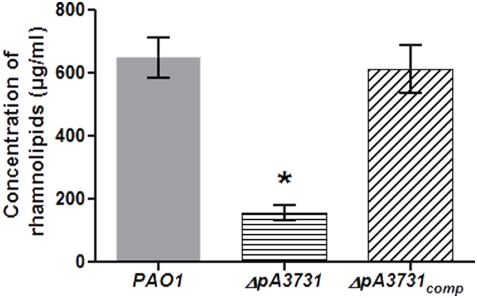
Influence of the mutation on rhamnolipid production. PAO1 (Grey); *ΔpA3731* (Horizontal line); *ΔpA3731*_comp_ (Oblique line). Bars: SEM (n = 10); *, P≤0.05.

Since swarming of *P. aeruginosa* is dependent on rhamnolipid production [8], we evaluated the impact of the mutation on this biosynthesis. Analyses (Fig. 5) showed that mutation on the gene *pA3731* resulted in a decrease in the rhamnolipid production. There again, complementation of the mutant by the *pA3731* gene reversed this phenotype.

### Infection assays

**Figure 6:**
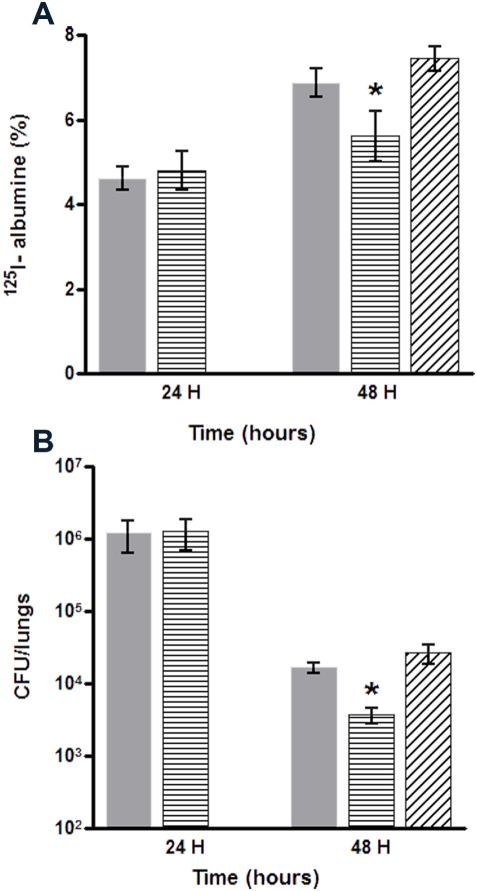
Infection assays. (A) Alveolar capillary barrier permeability and (B) lung bacterial clearance, after 24 and 48 h of infection. Mice were infected with 5×10^6^ CFU of *P. aeruginosa*. The number of viable bacteria remaining in the lungs was counted 48 h after the infection. PAO1 (Grey); *ΔpA3731* (Horizontal line); *ΔpA3731*_comp_ (Oblique line). Bars: SEM (n = 10); *, P≤0.05.

It has been shown that both motility and secreted products such as rhamnolipids are involved in *P. aeruginosa* dependent virulence. Virulence can be explored by different methods evaluating either host response like the lung bacterial load which reflects the capacity of the organism to eliminate the pathogen, or the alveolar permeability which measures the consequences of the injury. To evaluate the clinical consequences of the mutation on the *pA3731* gene, we used an *in vivo* model of acute pneumonia in which the parental or the mutated strains are injected intratracheally to mice. Experiments showed that bacterial growth of the mutant was affected during the acute phase of infection and its virulence attenuated (Fig. 6). Whereas no significant difference was observed after 24 h of infection, lung permeability (A) and bacterial load (B) were significantly lower after infection for 48 h with the mutant than with the parent strain. The complementation of the mutated strain was associated with a full recovery of the virulence reflected by these parameters.

### Characterization of the bac gene cluster

**Figure 7:**
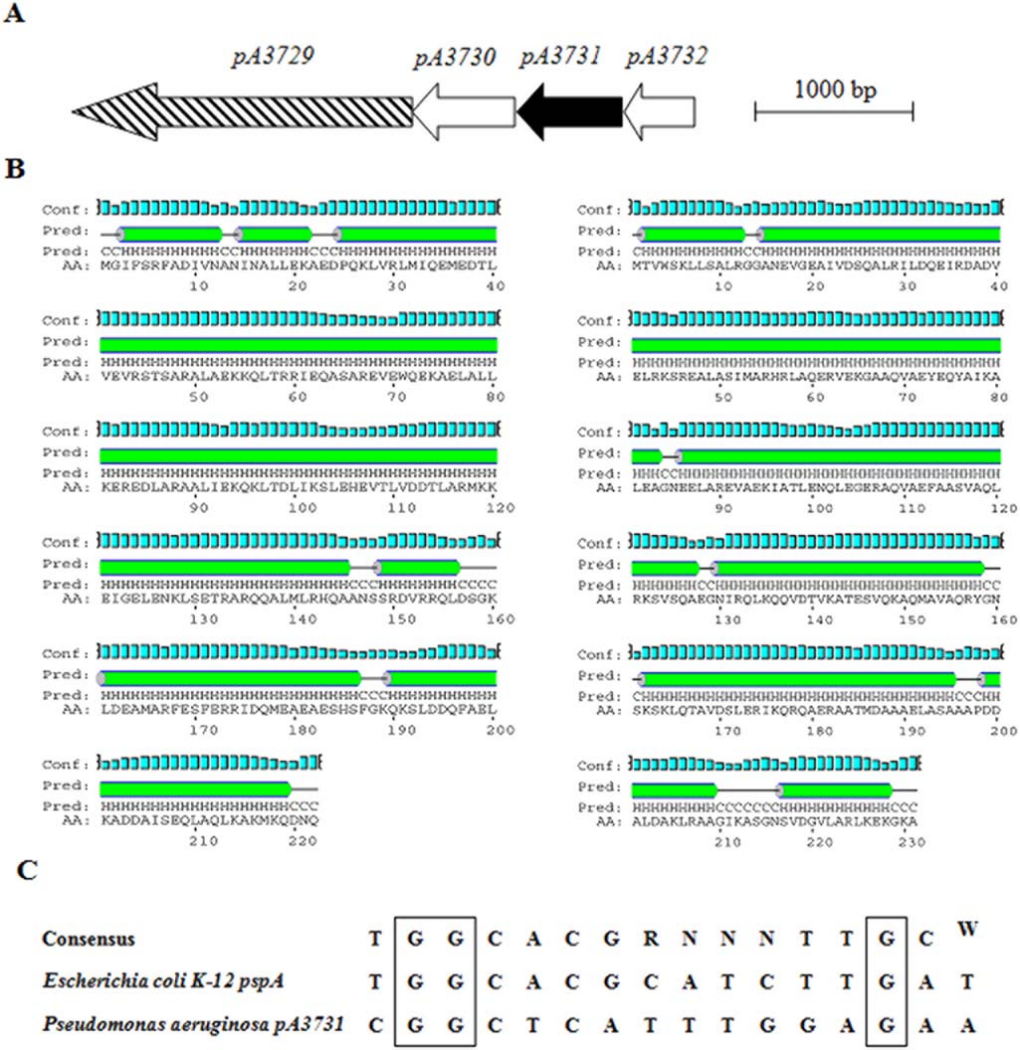
Bioinformatics analyses. A, organization of the *bac* gene cluster. Gene encoding an outer membrane protein is represented as a stripped arrow, genes encoding proteins exhibiting transmembrane domains are represented as white arrows and gene encoding a cytoplasmic protein is represented as a black arrow. The numbers above each gene correspond to the genome annotation (http://www.pseudomonas.com ); B, comparison of the secondary structures of PA3731 and PspA proteins, obtained by PredictProtein. Pred, predicted secondary structure; helix (In green); coil (Black line); conf (confidence of prediction, in blue); AA, target sequence; C, RpoN-binding sites of *E. coli pspA* and *P. aeruginosa bac*. The RpoN-binding sites of *pspA* promoters have a nonconsensus −12 dinucleotide. The core of the RpoN-binding site consensus sequence is shown at the top, with the highly conserved −24 and −12 dinucleotides overlined. Sequence data were obtained from the website pseudomonas.com (http://www.pseudomonas.com).

The availability of the *P. aeruginosa* PAO1 genome sequence (http://www.pseudomonas.com) allowed to identify the gene cluster associated with *pA3731*. The gene belongs to a cluster of 4 genes (*pA3729*–*pA3732*) coding for unknown proteins (Fig. 7A). Comparison of the protein sequence of PA3731 on databases showed no homology with other proteins. However, its predicted secondary structure showed high similarity with that of the Phage shock Protein A (PspA) (Fig. 7B). Analysis of the DNA sequence upstream from the *pA3732* gene allowed to identify a putative RpoN-binding site (Fig. 4C). The presence of a signal peptide cleavage site of an outer membrane protein in amino acid sequence is predicted for protein PA3729 (Mw 76 kDa) and transmembrane domains for proteins PA3730 (Mw 23 kDa) and PA3732 (Mw 16 kDa). No ATP-binding cassette (ABC) was found in protein sequences. We named this new system *bac* for biofilm-associated cluster.

### Heat and osmotic shocks

**Figure 8:**
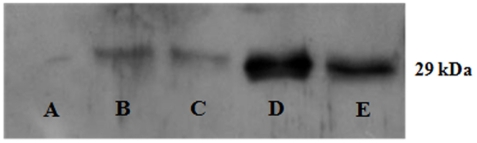
Expression of PA3731 under different growth conditions. Lane A, control (Δ*pA3731* grown at 37°C in LB broth); lane B, PAO1 grown at 37°C in LB broth; lane C, temperature shift from 37°C to 50°C; D, 20% saccharose, E, 0.2 M NaCl. Samples were loaded at 20 µg of proteins per lane. Proteins were electrotransferred on nitrocellulose membrane and assayed against an antiserum raised against the protein PA3731.

Since PspA protein is synthesized in response to heat and osmotic shocks [9], the effect of heat and osmotic shocks on the amount of PA3731 was investigated by using an immunoassay. Proteins of *P. aeruginosa* were separated on a SDS-PAGE and assayed against an antiserum raised against PA3731. As shown Fig. 8, PA3731 synthesis was induced upon hyperosmotic shocks but not after a shift from 37°C to 50°C.

## Discussion

Considering literature data, the biofilm phenotype of *P. aeruginosa* appears regulated more at the translational and perhaps post-translational levels than at the transcriptional level, as highlighted by the discrepancies between microarray and proteomic experiments. Whereas a transcriptome analysis led to the conclusion that only 1% (i.e., 73 genes) of *P. aeruginosa* genes showed differential expression in planktonic and biofilm cells [10], protein-based approaches suggested that a large number of proteins are differentially regulated during biofilm development [6], [11]. In addition to these global approaches, some investigations have characterized bacterial determinants involved in the formation and maturation of biofilms. Many of them are involved in bacterial motility. *P. aeruginosa* can perform three different types of cell motility: flagellum mediated “swimming”, type IV-pilus dependent “twitching” and a complex coordinated multicellular migration called “swarming” [12]–[14]. It has been shown that swarming of *P. aeruginosa* is not only dependent on flagella but also on type IV pili and rhamnolipids. Among surface-associated organelles described as important in biofilm formation, are flagella [3], [13], type IVa [3], [15] and type IVb pili [16]. Putative fimbrial structures, named cup for chaperone-usher pathways, have been also identified as playing a role in adhesion [4], [17].

The present work identified a new protein system of *P. aeruginosa* which is involved in both the interaction of the bacterium with surfaces (biotic and abiotic) and its virulence. Though we cannot categorically rule out a growth abnormality of sessile-cells (which is very difficult to measure), adhesion assays performed in PBS demonstrates that the decrease in biofilm formation exhibited by the mutant reflects an alteration in its ability to adhere on surfaces. All complementation assays allowed to recover wild-type phenotypes, demonstrating that the mutant features were only due to the disruption of the *pA3731* gene by the transposon. This alteration in the biofilm formation ability was accompanied by a decrease in bacterial swarming and rhamnolipid production. These results agree with literature data: biosurfactants as rhamnolipids which lower the surface tension facilitate the spreading of bacteria on semisolid surfaces [8]. The role of swarming in biofilm development [3], adhesion on mammalian cells and virulence [14] is well documented. In the later part of the study, we evaluated the clinical consequences of this mutation. In this aim, we used an *in vivo* model of acute pneumonia in which the parental or the mutated strains are injected intratracheally to mice. Two parameters were measured, i.e., alveolar permeability and bacterial load. The permeability reflects the barrier function of the alveoli capillary interface. This measurement is proportional to the extent of the injury in pneumonia and is measured by the leak of a vascular tracer (radiolabeled albumin) in the lung. The bacterial load quantifies the ability of mice to clear the bacteria from their infected lungs. We did not observe any difference in permeability and bacterial load between the mutant and parental strains after infection for 24 h. However, after 2 days, the mutant strain was associated with a significant decrease in both lung colonization and acute lung epithelial damage that was restored by complementation. These data pointed out the role of the PA3731 protein in *P. aeruginosa* virulence.

Like the phage-shock protein A (PspA), the predicted PA3731 protein contains a coiled-coil comprising 88% of the protein length. Proteins with coiled-coiled regions as extensive as those of PA3131 and PspA are relatively unusual in prokaryotes [18]. PspA is a hydrophilic 25.3 kDa polypeptide that is the product of the first gene of the *psp* system. This system is conserved in many different bacteria. Psp systems of *E. coli* and *Yersinia enterocolitica* have six proteins in common: PspA, B, C, D, F and G [19]. A speculative model for the Psp response systems has been recently proposed [19]. In this model, PspA binds to Psp F (a member of the enhancer-binding protein family of transcriptional regulators [20]) and prevents it from activating transcription. One or both inner membrane proteins, i.e., PspB and PspC, probably sense an inducing condition and interact with PspA, which frees PspF to activate transcription of the *pspA* operon. As a consequence, the concentration of PspA increases dramatically in comparison to the other Psp proteins. The *psp* operon is highly induced by the infection of *E. coli* with bacteriophage, by hyperosmotic and heat shocks [21]. This induction is caused by the synthesis of a single filamentous phage protein (protein IV) [21] which is a member of the secretin family of proteins found in a variety of bacterial secretion systems [22]. Synthesis of PspA protein is induced by the mislocalization of some outer membrane proteins, especially secretins (reviewed by Model et al [23].

While much is known about the induction and regulation of the Psp response, its physiological role remains questionable. *E coli* cells in which *psp* operon is disrupted are unable to maintain their proton-motive force, export proteins by the sec and tat pathways, and survive in stationary phase at alkaline pH [24]. *Y. enterocolitica pspC* mutant has a severe virulence defect in animal [9], [25]. The Psp system was induced in *E. coli* biofilm cells [26] and in *Salmonella enterica* during macrophage infection [27].

The question now concerns the physiological function of the locus *pA3729*–*pA3732*. We cannot yet answer this question. The phenotypes observed with the Δ*pA3731* mutant share some similarities with those described for *psp* mutants. Transcription of the Psp operon is dependent on σ^54^ [28], a sigma factor that plays a role in the architecture of *P. aeruginosa* biofilm via probably regulation of the quorum sensing [29]. The most highly conserved positions of the RpoN-binding site consensus are the GG and GC dinucleotides of the −24 and −12 regions. However, the C at position −12 has been replaced with A or T in RpoN-binding sites of many *pspA* homologs. Therefore, we analyzed the DNA sequence upstream from the *pA3732* gene and we could identify a putative RpoN-binding site. These data and the overproduction of PA3731 by hyperosmotic stresses suggest that the *pA3729*–*pA3732* locus is a *psp* regulon. However some obvious differences are observed between this locus and other Psp systems. Thus, all Psp systems described to date are encoded by two unlinked loci [24], which is not the case for the *pA3729–pA3732* cluster. Proteins PA3730 and PA3732 are predicted to be inner membrane proteins as PspBCD but there is no homology between them, and they exhibit molecular masses much higher than those of PspBCD that are small polypeptides (8, 13 and 8 kDa, respectively). PA3729 is predicted to be located in the outer membrane but this location has to be confirmed in the future. The *Pseudomonas* genome database (http://www.pseudomonas.com ) indicates that *pA3731* clusters with *yjfI* gene (b4182) of *E. coli* K12. However, the genetic organisation around this gene is very different from that of *pA3731* and no RpoN-binding site is present. The absence of impact of a temperature shift from 37 to 50°C on PA3731 production confirms differences between these two systems. Considering all these data, we named this new *P. aeruginosa* system *bac* for biofilm-associated cluster. To control the presence of this system in clinical *P. aeruginosa* strains, we amplified the *pA3731* gene by PCR in CF isolates. Results demonstrated the presence of the gene in all tested strains (data not shown).

In summary, infections with *P. aeruginosa* are a major problem for human health. Consequently, a better understanding of the adhesive mechanisms used by this bacterium to colonize tissues and abiotic surfaces is crucial to fight against the infection process. The present work reveals the involvement of a new protein system in the biofilm forming ability and virulence of the bacterium. Identifying the function of the *bac* system obviously represents a stimulating challenge that might open new avenues to treat infections caused by this important opportunistic pathogen.

## Materials and Methods

### Bacterial strains and growth conditions

Bacterial strains and plasmid used are listed in Table 1. *P. aeruginosa*, strain PAO1 and *ΔpA3731* mutant, were obtained from the mutant library available at the UWGC (University of Washington Genome Center). PAO1 was used as receptive strain of transposon IS*phoA*/hah (4.83 Kb) which derivates from the IS 50 L of the Tn*5* transposon. The transposition was generated by the method of Bailey & Manoil [30]. The complementary strain (*ΔpA3731*_comp_) was obtained by chemical transformation (see below) with the pMMB67-HE14 plasmid containing the *pa3731* gene.

**Table 1:** Bacteria and plasmid used in this study.

Strains	Description*	Source
PAO1	Wild type	UWGC
Δ*pA3731*	IS*phoA*/hah insertion::tet^R^	UWGC
Δ*pA3731*_comp_	Δ*pA3731* complemented with pMMB67 -HE14 expressing *pA3731*,amp^R^, tet^R^	This study
Plasmid		
pMMB67-HE14		Ball**

* amp^R^, ampicillin resistant, tet^R^, tetracyclin resistant.

** G. Ball, UPR CNRS 9027, IBSM, Marseille, France.

CF strains were obtained from patients hospitalized at the Charles Nicolle Hospital in Rouen (France).

All bacterial precultures were performed in Mueller Hinton Broth (MHB, Difco). According to the experiment (see below), Luria-Bertani (LB, Difco) or Brain Heart Infusion (BHI, Difco) broths were used. Antibiotics (carbenicillin and tetracyclin) were used at the concentration of 50 µg/ml.

### DNA manipulations and transformations

The good insertion of the transposon IS*phoA*/hah in the gene *pA3731* was controlled by PCR. Primers (forward: 5′-GGGAGGACACCATCATGACGG TATG-3′; reverse: 5′-TGTCGCTCTATCCCTGAAAAACGGG-3′) were designed to amplify the wild type gene region from upstream to downstream intergenic region (PCR product: 696 pb). For the mutant, primers (forward: 5′-GGGAGGACACCATCATGACGGTATG-3′; reverse: 5′-CGGGTGCAGTAATATCGCCCT-3′) were used to amplify the intergenic sequence to a part of the transposon sequence (PCR product: 623 pb). PCR was performed for 30 cycles of 5 min at 95°C, 30 s at 95°C, 30 s at 55°C and 1 min per Kb at 72°C.

A complemented strain (Δ*pA3731*_comp_) was constructed by inserting a wild-type copy of *pA3731* in the *pA3731* mutant strain. Plasmid pMMB67-HE14 was extracted from *Escherichia coli* (one Shot^R^ OmniMAX™-T1^R^Chemically Competent *E. coli* from Invitrogen) with GenElute™Plasmid MiniprepKit (Sigma-Aldrich) according to the manufacturer's instructions. For DNA amplifications, oligo-nucleotides (forward: 5′-ACAAGTTTGTACAAAAAAGCAGGCT-3′; reverse: 5′-ACCACTTTGTACAAGAAAGCTGGGT-3′) were purchased from Sigma-Proligo. DNA was introduced into the Δ*pA3731* strain. Electro-competent cells were prepared according to the protocol described by Choi *et al* [31].

Cells were grown overnight in 6 ml of LB medium with 50 µg/ml tetracyclin. Cells were equally distributed into 4 micro-centrifuge tubes and harvested by centrifugation at room temperature for 1–2 min at 14,000 g. One hundred µl of electro-competent cells was mixed with 50 ng of plasmid and transformation was performed by thermal shock (incubation at 42°C for 50 s followed by an incubation at 4°C for 10 min).

#### Planktonic growth kinetics

Free-cell cultures were performed in 250 ml Erlenmeyer flasks containing 100 mL of LB broth. Flasks were inoculated at 10^7^ CFU/mL and incubated at 37°C. The cell population was monitored by optical density measurements at 546 nm (OD 546) (Genesys 2PC spectrophotometer, Spectronic Instruments, Inc, Rochester, NY . Experiments were performed in triplicate. Results were expressed as mean±standard error of the mean (SEM).

#### Quantification of CV-stained attached cells

Tests were adapted from the method described by O'Toole & Kolter [3]. Bacteria were grown for 24 h in LB broth at 37°C in microtiter dishes. Unattached cells were removed by rinsing the microdishes thoroughly with water, and attached cells were subsequently stained by incubation with 0.5% CV for 20 min. CV was then solubilized by adding 1 mL of ethanol and the OD of the solution measured at 570 nm . All experiments were performed at least in triplicate.

### BioFilm Ring test®

In addition to the CV-test, we evaluated the biofilm forming ability of the bacterial strains by the BioFilm Ring Test® [7]. The device was kindly provided by the Biofilm Control Company (St Beauzire, France). The assay was carried out in modified polystyrene 96-wells microtiter plates as described [7]. Wells containing LB broth inoculated with a bacterial suspension and magnetic beads were placed onto a magnetic block test. After magnet contact, free beads were attracted in the centre of the bottom of wells, forming a black spot, while beads blocked by the biofilm remained in place. Images of each well before (I_0_) and after (I_1_) magnetization were compared with the BioFilm Control® software that gives a BioFilm Indice (BFI). A high BFI (>7) value corresponds to a high mobility of beads under magnet action (i.e. control wells) while a low value (<2) corresponds to a full immobilization of beads (positive biofilm). Each experiment was realized in triplicate

### Adhesion on A549 cell line

Bacterial adherence tests were carried out with the human A549 pneumocyte cell-line (ATCC CCl 165) according to the protocol described by Di-Martino et al [32]. Monolayers of epithelial cells were grown at 37°C in HAM F12 medium (Eurobio, les Ulis , France) containing 10% (v/v) fetal calf serum, 2 mM glutamine, and antibiotics (200 U of penicillin and 50 mg of streptomycin per liter, on 24-wells Falcon tissue culture plates (Becton, Dickinson Labware, Oxnard, CA). Confluent cells were washed with phosphate-buffered saline (PBS, pH 7.2) and incubated for 3 h at 37°C in the presence of bacteria (initial cell concentration in the medium, 10^7^ CFU/ml). After 5 washings with PBS, cells were fixed in methanol, stained with 20% (v/v) Giemsa, and examined microscopically. An adhesion index representing the average number of bacteria per cell was determined (examination of 100 cells). Adhesion was considered positive if the adhesion index was >1 [32].

#### Adhesion assay in phosphate buffer

The assay was adapted from the CV test (see above). Bacteria (initial cell concentration, 10^7^ Colony Forming Units (CFU) /ml) were incubated for 0.5 h in PBS at room temperature in microtiter dishes. Unattached cells were removed by rinsing the microdishes thoroughly with water, and attached cells were subsequently harvested by scraping and resuspended in PBS. Appropriate dilutions of the cell suspension were plated out on LB agar plates. Colonies were counted after incubation of the plates for 24 h at 37°C. All counts were performed in triplicate. Results were expressed in CFU per cm^2^ ±SEM (n = 10).

### Motility assays

Bacteria were stab inoculated with a needle into the bottom of LB agar medium (1.5, 0.3 or 0.6% agar for twitching, swimming or swarming tests, respectively, average inoculation depth 3 mm). Plates were incubated at 37°C for 24 h. For swimming and swarming assays, the migration zones were directly measured. For twitching tests, the zones were measured after staining with 0.5% Crystal Violet (CV, Sigma-Aldrich). Results were expressed as mean±standard error of the mean (SEM).

### Semiquantitative determination of rhamnolipids production

Tests were adapted from the method described by Koch et al [33]. The orcinol assay was used to assess the amount of rhamnolipids in samples [34]. After a culture of 48 h, 2 ml of the culture supernatant was extracted twice with 1 ml of diethyl ether. The pooled ether fractions were evaporated to dryness and the remainder was dissolved in 100 µl distilled water and mixed with 100 µl of 1.6% orcinol, and 800 µl of 60% sulfuric acid. After heating for 30 min at 80°C in the dark, the samples were cooled for 3 h at room temperature in the dark and the absorbance at 421 nm (A_421_) was measured. Rhamnolipids concentrations were calculated by comparing A_421_ values with those obtained for rhamnose standards between 0 and 1000 µg/ml, assuming that 1 µg of rhamnose corresponds to 2.5 µg of rhamnolipids.

### P. aeruginosa-induced lung injury

Male BalB/c mice (20–25 g) purchased from Charles River Laboratories (Domaine des Oncins, L'arbresle, France) were housed in a pathogen-free unit of the Lille University Animal Care Facility and provided with food and water *ad lib*. All experiments were performed with the approval of the Lille Institutional Animal Care and use committee. *P. aeruginosa* was incubated in 5 ml of LB broth at 37°C for 16 h in a shaking incubator. After centrifugation at 3,000 g for 10 min, the bacterial pellets were washed two times in isotonic saline solution and resuspended in isotonic saline solution to yield an appropriate number of CFU per ml (determined by OD measurement at 600 nm). Acute injury was produced according to the method by Pennington and Ehrie. Mice were briefly anesthetised with inhaled sevoflurane (Ultame, Abbot Laboratories, Abbot Park, Illinois, USA) and placed in a supine position at an angle of approximately 30°. For each mouse, 50 µl of a bacterial inoculum was instilled into the lungs with a gavage needle (modified animal feeding needle, 24 G, Popper & Sons, Inc., New Hyde Park, NY) inserted into trachea via the oropharynx. The proper insertion of the needle was confirmed by observing the movement of the solution inside the syringe during the animal's respiratory efforts.

### Measurement of alveolar capillary barrier permeability after P. aeruginosa instillation

The method used measures ^125^I-albumin injected intraperitoneally as a vascular protein tracer in the extravascular spaces of the lungs. The album in flux across the barrier was calculated using the Permeability Index, previously described for rats [35]. Briefly, 0.5 ml of ^125^I-labelled albumin was injected intraperitoneally. Two hours later, exsanguination was performed after i.p. injection of pentobarbital sodium. The lungs were removed through a sternotomy, and blood radioactivity and haemoglobin (Hb) concentration were measured. Lung weighing and radioactivity counts were performed before homogenization and centrifugation. The supernatant Hb content was measured. Blood and lung homogenate samples were placed at 80°C for 3 days to determine the wet-to-dry weight ratio (W/D). The permeability index (PI) was calculated as follows: PI = [(Radioactivity count _lungs_−(Radioactivity count _intravascular blood per gram of blood_×Q_B_)) / (Radioactivity count _intravascular blood per gram of blood_×Weight _mouse_)]×100, where: Q_B_ is the weight of intrapulmonary blood and was calculated. Q_B_ = (Weight _lung+water_×Hb concentration _supernatant_×Water ratio _homogenate_×1.039) / (Hb concentration _blood_×Water ratio _blood_).

### Pulmonary bacterial load

Lungs were removed after 24 or 48 h of infection and homogenized in 0.9 ml of sterile isotonic saline. Viable bacteria were counted by plating 0.1 ml of serial dilutions of the homogenates on agar plates for 24 h at 37°C.

### Immunization

The *pa3731* gene was cloned in an *Escherichia coli* overexpressin*g* vector and transformed into *E. coli* BL21DE3-B834. Recombinant protein expression was performed by 0.5 mM IPTG induction for 2 h at 37°C. Protein PA3731 was purified by Ni-chelate chromatography (Qiagen). New Zealand White male rabbits (Charles River Laboratories) were immunized with PA3731 protein every 15 days over 5 months. In brief, 250 µg of protein (in 500 µl) were mixed with 500 µl of Freund complete adjuvant for the first injection then of incomplete Freund adjuvant for next injections and intradermally administered. Rabbits were bled one week after each boost and ELISA was used to monitor anti-PA3731 antibody titers in the serum. Animal experiments were performed with the approval of the Haute-Normandie region ethical committee for animal experimentation.

### Western blot analysis

*P. aeruginosa* proteins were separated on a 12% SDS-PAGE and electrotransferred to a nitrocellulose membrane (Sigma). The membrane was blocked by 5% nonfat milk in TTBS overnight at room temperature, followed by a single wash with TTBS. The membrane was incubated with the antiserum raised against PA3731 (1∶1,000) for 1.5 h. After six washes with TTBS, a peroxydase conjugated anti-rabbit antibody was added and the membrane was incubated for 2 h at room temperature. The membrane was washed six times and the reactivity was detected using an ECL Western blotting detection kit (Amersham Biosciences) and exposure to a Kodak film.

### Heat and osmotic shocks

Bacteria (10^7^ UFC/ml) were grown in LB at 37°C until an OD_545_ of 1.0 was reached. For heat shock (shift from 37°C to 50°C) bacteria were incubated for 10 min in 50°C water. For osmotic shock, cells were centrifuged for 10 min at 5000 rpm, resuspended in 50 ml of 20% saccharose or 50 ml of 0.2 M NaCl and incubated at 37°C for 10 min. After treatments, cells were recovered by centrifugation, resuspended in 50 ml of distilled sterile water and incubated for 10 min at 37°C under shaking. Finally, cells were collected by centrifugation and lysed in distilled water by a freezing/defrosting temperature shock (from −20°C to 20°C) followed by sonication.

### Protein secondary structure prediction

The comparison of the secondary structures of PA3731 and *E. coli* PspA was performed using PredictProtein, the service for sequence analysis accessible at http://www.predictprotein.org.

### Genome sequence analysis

DNA sequences were characterized using the PAO1 genome accessible at http://www.pseudomonas.com.

### Data analyses

All experiments were performed at least three times. Results were expressed as mean±standard error of the mean (SEM). Calculations were performed using graphic software (GraphPad Prism 5.0). Results were analysed using Fisher F- and Student's t-tests (with or without Welsh correction). Differences were considered significant if the P value was ≤0.05.

## References

[1] Costerton JW, Stewart P, Greenberg EP (1999). Bacterial biofilms: a common cause of persistent infections.. Science.

[2] Tummler B, Kiewitz C (1999). Cystic fibrosis: an inherited susceptibility to bacterial respiratory infections.. Mol Med Today.

[3] O'Toole G, Kolter R (1998). Flagellar and twitching motility are necessary for *Pseudomonas aeruginosa* biofilm development.. Mol Microbiol.

[4] Vallet I, Olson JW, Lory S, Filloux A (2001). The chaperone/usher pathways of *Pseudomonas aeruginosa*: identification of fimbrial gene clusters (*cup*) and their involvement in biofilm formation.. Proc Natl Acad USA.

[5] Vasseur P, Vallet-Gely I, Soscia C, Genin S, Filloux A (2005). The *pel* genes of the *Pseudomonas aeruginosa* PAK strain are involved at early and late stages of biofilm formation.. Microbiology.

[6] Vilain S, Cosette P, Hubert M, Lange C, Junter GA (2004). Comparative proteomic analysis of planktonic and immobilized *Pseudomonas aeruginosa* cells: a multivariate statistical approach.. Anal Biochem.

[7] Chavant P, Gaillard-Martinie B, Hébraud M, Bernardi T (2007). A new device for rapid evaluation of biofilm formation potential by bacteria.. J Microbiol Methods.

[8] Caiazza NC, Shanks RMQ, O'Toole GA (2005). SadB is required for the transition from reversible to irreversible attachment during biofilm formation by *Pseudomonas aeruginosa* PA14.. J Bacteriol.

[9] Darwin AJ, Miller VL (1999). Identification of *Yersinia enterocolitica* genes affecting survival in an animal host using signature-tagged transposon mutagenesis.. Mol Microbiol.

[10] Whiteley M, Bangera MG, Bumgarnes RE, Parsek MR, Teitzeil GM (2001). Gene expression in *Pseudomonas aeruginosa* biofilms.. Nature.

[11] Sauer K, Camper AK, Erhlich GD, Costerton JW, Davies DG (2002). *Pseudomonas aeruginosa* displays multiple phenotypes during development as a biofilm.. J Bacteriol.

[12] Mattick JS (2002). Type IV pili and twitching motility.. Annu Rev Microbiol.

[13] Rashid MH, Kornberg A (2000). Inorganic polyphosphate is needed for swimming, swarming, and twitching motilities of *Pseudomonas aeruginosa*.. Proc Natl Acad Sci USA.

[14] Sharma M, Anand SK (2002). Swarming: a coordinated bacterial activity.. Curr Scie.

[15] Klausen M, Heydorn A, Ragas P, Lambertsen L, Aes-Jorgensen A (2003). Biofilm formation by *Pseudomonas aeruginosa* wild type, flagella and type IV pili mutants.. Mol Microbiol.

[16] De Bentzmann S, Aurouze M, Ball G, Filloux A (2006). FppA, a novel *Pseudomonas aeruginosa* prepilin peptidase involved in assembly of type IVb pili.. J Bacteriol.

[17] Ruer S, Stender S, Filloux A, De Bentzmann S (2007). Assembly of fimbrial structures in *Pseudomonas aeruginosa*: functionality and specificity of chaperone-usher machineries.. J Bacteriol.

[18] Dworkin J, Javanovic G, Model P (2000). The PspA protein of *Escherichia coli* is a negative regulator of sigma(54)-dependent transcription.. J Bacteriol.

[19] Darwin AJ (2005). The phage-shock-protein response.. Mol Microbiol.

[20] Jovanovic G, Weiner L, Model P (1996). Identification, nucleotide sequence, and characterization of PspF, the transcriptional activator of the *Escherichia coli* stress-induced psp operon.. J Bacteriol.

[21] Brissette JL, Russel M, Weiner L, Model P (1990). Phage shock protein, a stress protein of *Escherichia coli*.. Proc Natl Acad Sci USA.

[22] Genin S, Boucher CA (1994). A superfamily of proteins involved in different secretion pathways in gram-negative bacteria: modular structure and specificity of the N-terminal domain.. Mol Gen Genet.

[23] Model P, Jovanovic G, Dworkin J (1997). The *Escherichia coli* phage-shock-protein (psp) operon.. Mol Microbiol.

[24] Kleerebezem M, Crielaard A, Tommassen J (1996). Involvement of stress protein PspA (phage shock protein A) of *Escherichia coli* in maintenance of the protonmotive force under stress conditions.. EMBO J.

[25] Darwin AJ, Miller VL (2001). The *psp* locus of *Yersinia enterocolitica* is required for virulence and for growth in vitro when the Ysc type III secretion system is produced.. Mol Microbiol.

[26] Beloin C, Valle J, Latour-Lambert P, Faure P, Kzreminski M (2004). Global impact of mature biofilm lifestyle on *Escherichia coli* K-12 gene expression.. Mol Microbiol.

[27] Green RC, Darwin AJ (2004). PspG, a new member of the *Yersinia enterocolitica* phage shock protein regulon.. J Bacteriol.

[28] Eriksson S, Lucchini S, Thompson A, Rhen M, Hinton JC (2003). Unravelling the biology of macrophage infection by gene expression profiling of intracellular *Salmonella enterica*.. Mol Microbiol.

[29] Thompson LS, Webb JS, Rice SA, Kjelleberg S (2003). The alternative sigma factor RpoN regulates the quorum sensing gene *rhlI* in *Pseudomonas aeruginosa*.. FEMS Microbiol Lett.

[30] Bailey J, Manoil C (2002). Genome-wide internal tagging of bacterial exported proteins.. Nat Biotechnol.

[31] Choi KH, Kumar A, Schweitzer HP (2006). A 10-min method for preparation of highly electrocompetent *Pseudomonas aeruginosa* cells: application for DNA fragment transfer between chromosomes and plasmid transformation.. J Microbiol Methods.

[32] Di-Martino P, Gagnière H, Berry H, Bret L (2002). Antibiotic resistance and virulence properties of *Pseudomonas aeruginosa* strains from mechanically ventilated patients with pneumonia in intensive care units: comparison with imipenem-resistant extra-respiratory tract isolates from uninfected patients.. Microb Infec.

[33] Koch A, Käppeli O, Fiechter A, Reiser J (1991). Hydrocarbon assimilation and biosurfactant production in *Pseudomonas aeruginosa* mutants.. J Bacteriol.

[34] Chandrasekaran EV, Bemiller JN, Whistler RL (1980). Constituent analyses of glycoaminoglycans.. Methods in carbohydrate chemistry.

[35] Robriquet L, Collet F, Tournoys A, Prangere T, Neviere R (2006). Intravenous administration of activated protein C in *Pseudomonas*-induced lung injury: impact on lung fluid balance and the inflammatory response.. Respir Res.

